# Patient‐Specific Lumped‐Parameter Model for Quantifying Vessel‐Specific Remodeling and Predicting Right Ventricular Function in Pulmonary Hypertension

**DOI:** 10.1002/cph4.70102

**Published:** 2026-01-26

**Authors:** Christopher G. Lechuga, Amirreza Kachabi, Mitchel J. Colebank, Claudia E. Korcarz, Farhan Raza, Naomi C. Chesler

**Affiliations:** ^1^ Edwards Lifesciences Foundation Cardiovascular Innovation and Research Center (CIRC) and Department of Biomedical Engineering University of California, Irvine Irvine California USA; ^2^ Department of Mathematics University of South Carolina Columbia South Carolina USA; ^3^ Department of Biomedical Engineering University of South Carolina Columbia South Carolina USA; ^4^ Department of Medicine‐Cardiovascular Division University of Wisconsin–Madison Madison Wisconsin USA

**Keywords:** hemodynamic simulation, lumped‐parameter modeling, pressure–volume loops, pulmonary hypertension, right ventricular function

## Abstract

**Purpose:**

Pulmonary hypertension (PH) is a heterogeneous disease with patient‐specific variability and vessel‐specific remodeling, which eventually lead to right ventricular (RV) failure. The gold standard for RV assessment—pressure–volume (PV) loop acquisition—is invasive and limited to specialized settings. This study aims to develop a patient‐specific lumped‐parameter model that quantifies vessel‐specific remodeling and simulates RV PV loops across PH phenotypes using routine clinical data.

**Methods:**

A lumped‐parameter model was calibrated using right heart catheterization and echocardiography data. Model agreement was assessed by *R* ^2^ values for pressure and flow goodness‐of‐fit, and model‐derived hemodynamic metrics were compared with clinical values. A dimensionality reduction approach was applied to investigate how well different PH phenotypes could be separated.

**Results:**

Across the cohort, the lumped‐parameter model showed good agreement with clinical data. Model‐derived vessel‐specific (pulmonary arterial, capillary, venular) parameters highlighted physiological distinctions among phenotypes. Predicted RV PV loops revealed phenotype‐specific differences in right ventricular volumes, pressures, and stroke work. The linear discriminant analysis (LDA) demonstrated qualitative separability, indicating that model‐derived, nonmeasurable features offer additional discriminatory information.

**Conclusion:**

Our results demonstrate that lumped‐parameter models can be calibrated to clinical data to quantify vessel‐specific remodeling and simulate RV pressure–volume dynamics to provide useful information for distinguishing among different PH phenotypes. This underscores the potential of computational models as noninvasive, clinically feasible tools for assessing in‐depth pulmonary vascular and RV function in PH.

## Graphical abstract

Personalized, patient‐specific biophysical model enabling mechanistic assessment of right ventricular (RV) function as well as robust differentiation of pulmonary hypertension (PH) subtype from routine clinical measurements of pulmonary arterial pressure and flow.

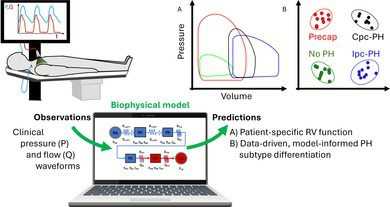

## 1 Introduction

Pulmonary hypertension (PH) is a progressive and life‐threatening disease associated with high mortality rates (Chang et al. 2022; Lin et al. 2022; Rose et al. 2019; Humbert et al. 2022; Taniguchi et al. 2019), affecting an estimated 70–80 million people globally, with a prevalence that is rising annually (Mocumbi et al. 2024; Thiwanka Wijeratne et al. 2018). PH can develop from a variety of underlying conditions including chronic thromboembolic disease in the pulmonary arteries (CTEPH), left heart disease (PH‐LHD), and idiopathic pulmonary arterial hypertension (PAH) (Humbert et al. 2022; Simonneau et al. 2019). PH also can affect different compartments of the pulmonary vasculature—the arteries, veins, or both—leading to distinct PH phenotypes, including pre‐capillary PH (arterial compartment), isolated postcapillary PH (Ipc‐PH; venous compartment), and combined pre‐ and postcapillary PH (Cpc‐PH; both compartments) (Humbert et al. 2022; Simonneau et al. 2019). Progressive PH of any type results in increased pulmonary arterial pressure and right ventricular (RV) afterload, causing RV dysfunction and eventually RV failure (Humbert et al. 2022; Simonneau et al. 2019; Rosenkranz et al. 2020). Early and accurate diagnosis of PH is critical for guiding appropriate treatment strategies; however, clinical assessment remains challenging due to the heterogeneity of the disease and its phenotypes (Humbert et al. 2022; Simonneau et al. 2019). Current diagnostic approaches, including right heart catheterization (RHC) and echocardiography, provide crucial data (Humbert et al. 2022; Simonneau et al. 2019), but the need for more accessible, efficient diagnostic tools in the clinical setting remains clear.

As PH progresses, RV dysfunction becomes a hallmark of disease severity and prognosis, making it key to detect as part of routine clinical evaluation (Nabeshima et al. 2024; Richter et al. 2021; Schmeißer et al. 2021a). Metrics derived from pressure‐volume (PV) loops are the gold standard for assessing RV function and its coupling with the pulmonary vasculature (Richter et al. 2021; Schmeißer et al. 2021a, 2021b; Brener et al. 2022). Real‐time, simultaneous measurements of RV pressure and volume throughout the cardiac cycle yield valuable metrics such as end‐systolic and end‐diastolic pressures and volumes (ESP, EDP, ESV, and EDV), and quantify myocardial energetics (oxygen consumption) with stroke work (SW, space within the loop) (Brener et al. 2022; Suga 1990; Seemann et al. 2019). These parameters are essential for characterizing RV contractility, afterload, and the work during contraction (Richter et al. 2021; Brener et al. 2022; Bellofiore et al. 2018). Despite their diagnostic value, PV loop acquisition requires specialized equipment not available in standard clinical practice (Brener et al. 2022). As a result, diagnostic use of PV loop metrics remains limited to research settings.

One approach to address this unmet gap is through computational models, which provide insights into cardiopulmonary disease that are impractical or infeasible to obtain through clinical measurements alone (Kachabi et al. 2025). In particular, models capable of simulating PV loops from more readily obtained data can help fill this unmet clinical need (Garber et al. 2022; Kheyfets et al. 2016; Tang et al. 2020; Keshavarz‐Motamed 2020; Kim et al. 2023). Lumped‐parameter models, which simplify the circulatory system into a few physiologically relevant components such as time‐varying elastance for the RV and Windkessel elements for the pulmonary vasculature, run quickly and have few modifiable parameters subject to uncertainty (Garber et al. 2022; Colunga et al. 2023; Colebank et al. 2024). These models offer a promising tool for predicting RV function across different PH phenotypes from routine clinical data, providing additional metrics to improve PH diagnosis and treatment planning. Moreover, these models can quantify vessel‐specific remodeling of the pulmonary arteries, capillaries, and veins, which are important clinically but not readily apparent from clinical measures.

Thus, the aim of this study is to develop and calibrate participant‐specific lumped‐parameter models for different PH phenotypes, quantify vessel‐specific remodeling, simulate RV PV loops, and provide hemodynamic metrics that are not captured by current gold‐standard procedures using standard clinical measurements. We anticipate that this approach can provide insight into PH phenotype‐specific pathophysiology and aid in clinical management of PH.

## 2 Materials and Methods

All participants provided informed consent in agreement with guidelines approved by the UW‐Madison institutional review board (IRB ID: 2019‐1184). The study complied with the guidelines of the Declaration of Helsinki and was overseen by an independent safety and monitoring board.

Materials used, data gathered, and methods applied in this research will be made available upon reasonable request in writing for purposes of reproduction and/or replication.

### 2.1 Study Population

Twenty‐seven adult participants (16 female, 11 male) were referred to the clinical setting for invasive cardiopulmonary exercise testing (iCPET) to evaluate unexplained dyspnea. Inclusion criteria included: New York Heart Association (NYHA) functional class II–III, evidence of PH on echocardiogram (PA systolic pressure > 35 mmHg), and ability to participate in exercise testing. Exclusion criteria included: age > 80 years, body mass index > 40.0 kg/m^2^, NYHA I or IV, contraindications to MRI, active cardiac problems (angina or unstable arrhythmias), implanted pacemaker or defibrillator, left ventricular ejection fraction < 50%, lung parenchymal disease requiring supplemental oxygen, end‐stage renal disease or chronic kidney disease (creatinine > 2.0 mg/dL), any acute illness, or hospitalization within the prior 3 months.

All participants were diagnosed with specific PH phenotypes based on gold standard parameters (mean pulmonary arterial pressure (mPAP), pulmonary arterial wedge pressure (PAWP), pulmonary vascular resistance (PVR)) via RHC, per 2019 ESC/ERS guidelines (Simonneau et al. 2019). Of the 27 participants enrolled, two had uninterpretable data due to motion artifact and were excluded. The remaining 25 enrolled participants were then categorized as: pre‐capillary PH (including PAH [*n* = 3] and CTEPH [*n* = 2]), Ipc‐PH (*n* = 12), Cpc‐PH (*n* = 5), and those without PH (No PH, *n* = 3) (Simonneau et al. 2019). Participants with either Ipc‐PH or Cpc‐PH had PH due to heart failure with preserved ejection fraction (PH‐HFpEF). Clinical hemodynamics and diagnoses for all subjects are provided in Table 1. Pulmonary vascular impedance and wave mechanics in these participants have been previously reported (Raza et al. 2022; Lechuga et al. 2025a, 2025b).

**TABLE 1:** Clinical hemodynamics of all subjects.

Subjects	Cohort	Sex	Age	HR (bpm)	CO (L/min)	PAWP (mmHg)	mPAP (mmHg)
#1	Pre‐cap	F	75	68	4.0	12	38
#2	Pre‐cap	F	70	71	3.4	12	26
#3	Pre‐cap	F	34	72	6.7	15	38
#4	Pre‐cap	M	67	60	4.8	11	40
#5	Pre‐cap	F	71	85	4.6	14	22
#6	Ipc‐PH	M	71	56	4.7	10	15
#7	Ipc‐PH	M	69	77	4.8	13	23
#8	Ipc‐PH	M	66	64	4.9	20	31
#9	Ipc‐PH	M	80	74	5.1	16	28
#10	Ipc‐PH	M	56	60	5.8	18	32
#11	Ipc‐PH	F	67	59	5.8	20	32
#12	Ipc‐PH	M	67	75	9.3	22	38
#13	Ipc‐PH	F	69	81	7.8	16	25
#14	Ipc‐PH	F	70	83	6.3	18	30
#15	Ipc‐PH	F	77	76	5.5	14	25
#16	Ipc‐PH	M	58	74	6.8	16	26
#17	Ipc‐PH	F	52	81	7.6	11	17
#18	Cpc‐PH	F	76	55	3.8	20	44
#19	Cpc‐PH	F	78	62	3.0	24	46
#20	Cpc‐PH	F	77	76	4.5	16	32
#21	Cpc‐PH	F	77	69	6.8	20	46
#22	Cpc‐PH	F	84	84	2.7	20	45
#23	No PH	F	68	88	6.0	8	18
#24	No PH	F	74	80	4.3	11	20
#25	No PH	F	65	92	8.1	12	22

Abbreviations: CO, cardiac output; HR, heart rate; mPAP, mean pulmonary artery pressure; PAWP, pulmonary arterial wedge pressure.

### 2.2 Clinical Data: Right Heart Catheterization and Echocardiogram

**FIGURE 1:**
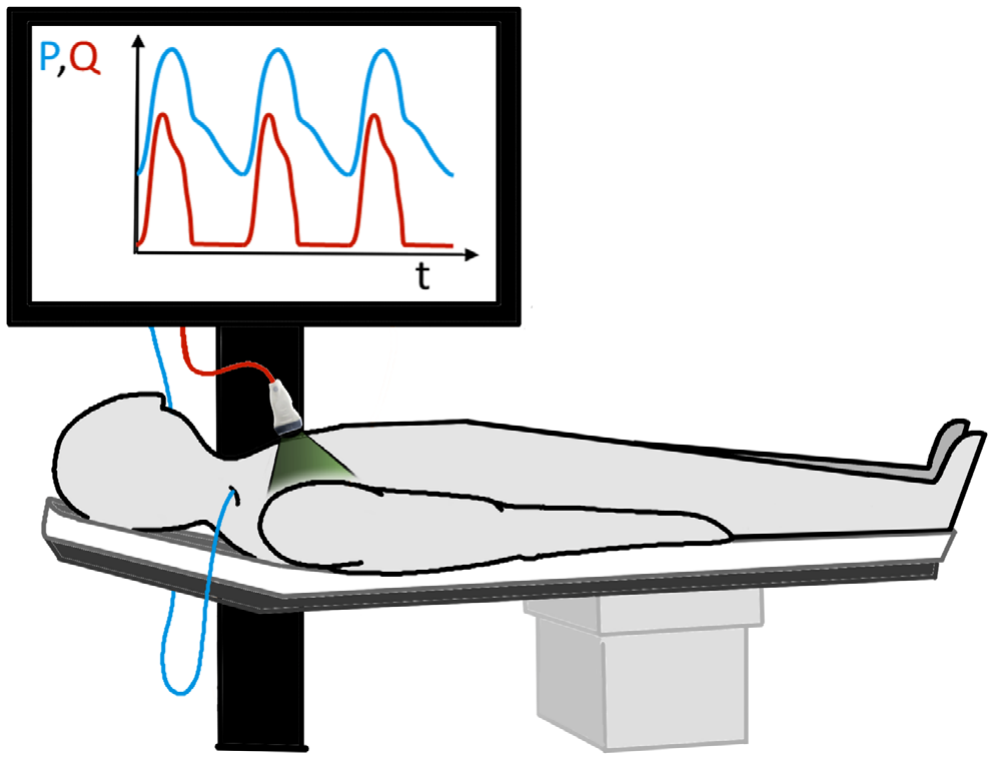
Schematic representation of right heart catheterization, and echocardiography. In a semi‐recumbent position, pulmonary artery pressure was measured via RHC, RVOT flow was measured using pulsed‐wave Doppler echocardiography. Pulmonary artery pressure (PAP) and RVOT flow (*Q*_RVOT_) wave forms were used for parameter estimation. *P*, pressure; RHC, right heart catheterization; RVOT, right ventricular outflow tract.

All participants underwent simultaneous RHC and echocardiography in a semi‐recumbent position (Figure 1) (Kozitza et al. 2022). Pulmonary artery pressure (PAP) was measured using a high‐fidelity 4Fr Millar catheter and velocity‐time integral (VTI) in the right ventricular outflow tract (RVOT) using pulsed‐wave Doppler (PWD) as previously described (Raza et al. 2022; Lechuga et al. 2025a; El Shaer et al. 2024). As an approximation of flow through the pulmonary valve, RVOT flow (*Q*_RVOT_) was calculated by multiplying the VTI by the RVOT cross‐sectional area, derived from echo‐based RVOT diameter measurements; only data with 3–5 high‐quality beats were included, while data points with limited echocardiographic windows (e.g., poor‐quality RVOT signal or inaccurate sample volume location) were excluded. To ensure alignment of pressure and flow signals from clinical data, we used a custom MATLAB program as previously reported (Lechuga et al. 2025a).

### 2.3 Mathematical Model Details

**FIGURE 2:**
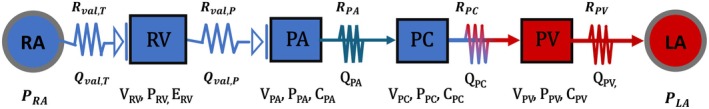
Schematic representation of the lumped‐parameter model. The model incorporates six compartments representing the RA, RV, PA, PC, PV, and LA with resistive elements ($R_{\mathrm{val},T}$, $R_{\mathrm{val},P}$, $R_{\mathrm{PA}}$, $R_{\mathrm{PC}}$, $R_{\mathrm{PV}}$) and compliant elements ($C_{\mathrm{PA}}$, $C_{\mathrm{PC}}$, $C_{\mathrm{PV}}$) as indicated. The RV chamber is modeled using a time‐varying elastance function ($E_{\mathrm{RV}}\left(t\right)$) to capture systolic and diastolic pressure–volume relationships. Pressures (P) and flows (Q) are defined for each compartment, with fixed boundary conditions at $\;P_{\mathrm{RA}}$ and $\;P_{\mathrm{LA}}$ derived from literature and the minimum of the PAWP and diastolic PAP, respectively. C, compliance; $C_{\mathrm{PA}}$, compliance of the pulmonary artery; $C_{\mathrm{PC}}$, compliance of the pulmonary capillaries; $C_{\mathrm{PV}}$, compliance of the pulmonary veins; $E_{\mathrm{RV}}$, right ventricular elastance; LA, left atrium; P, pressure; PA, pulmonary artery; PAP, pulmonary artery pressure; PAWP, pulmonary arterial wedge pressure; PC, pulmonary capillaries; PV, pulmonary veins; $P_{\mathrm{LA}}$, left atrial pressure; $P_{\mathrm{RA}}$, right atrial pressure; *Q*, flow; *R*, resistance; RA, right atrium; $R_{\mathrm{PA}}$, resistance from the pulmonary arteries to the pulmonary capillaries; $R_{\mathrm{PC}}$, resistance from the pulmonary capillaries to the pulmonary veins; $R_{\mathrm{PV}}$ resistance from the pulmonary veins to the left atrium; RV, right ventricle; $R_{\mathrm{val},P}$, resistance from the right ventricle to the pulmonary artery across the pulmonic valve; $R_{\mathrm{val},T}$, resistance from the right atrium to the right ventricle across the tricuspid valve.

A lumped‐parameter model with four dynamic compartments, the RV, PA, pulmonary capillaries (PC), and pulmonary veins (PV), was used to simulate pulmonary hemodynamics and RV function (Figure 2). The right atrium (RA) and left atrium (LA) were modeled as constant, patient‐specific pressure sources (i.e., fixed inputs). The PA, PC, and PV compartments were modeled as 2‐element Windkessels with compliance ($C_{i}$) and resistance ($R_{j}$) where i = PA, PC, and PV and j = val,T, val,P, PA, PC, and PV. The tricuspid and pulmonic valves (val,T and val,P, respectively) were modeled as diodes, with val,T permitting unidirectional flow in the absence of losses during diastole and preventing flow during systole, and val,P functioning oppositely (Colunga et al. 2023). None of the patients included in this study had clinically significant (moderate or severe) pulmonic or tricuspid valve regurgitation, supporting the use of this idealized valve representation.

The pressure in the RV ($P_{\mathrm{RV}}$) was modeled using a linear, time‐varying elastance function ($E_{\mathrm{RV}}\left(t\right)$):

(1) $$P_{\mathrm{RV}}\left(t\right)=E_{\mathrm{RV}}\left(t\right)\left(V_{\mathrm{RV}}\left(t\right)-V_{0}^{\mathrm{RV}}\right)$$

where $E_{\mathrm{RV}}\left(t\right)$ varies between a minimum ($E_{\min}$) and maximum ($E_{\max}$) during the cardiac cycle, $V_{\mathrm{RV}}\left(t\right)$ is the dynamic volume (see Equation 2), and $V_{0}^{\mathrm{RV}}$ represents the RV volume at zero pressure, here set to 0 (mL). We employ the elastance curve from Colunga et al., which uses timing parameters $t_{\max}$ and $t_{\min}$ to define the systolic and diastolic phases (Colunga et al. 2023). The dynamics of each compartment are governed by the following relationships (see Figure 2 for inflow/outflow and upstream/downstream relationships):

1. Volume Conservation: the volume within any compartment is the difference between the inflow and outflow

(2) $$\begin{array}{c} \frac{{\mathrm{dV}}_{k}}{\mathrm{dt}}=Q_{\text{in}}-Q_{\text{out}} \end{array}$$

where k = RV, PA, PC, and PV; and $Q_{in}$ and $Q_{\text{out}}$ represent inflow and outflow, respectively.

2 Flow Dynamics: following an electrical circuit analog, flow is proportional to the pressure gradient over the vascular or valvular resistance

(3) $$\begin{array}{c} Q_{j}=\frac{P_{\mathrm{up}}-P_{\text{down}}}{R_{j}} \end{array}$$

where j = val,T, val,P, PA, PC, and PV; $P_{\mathrm{up}}-P_{\text{down}}$ is the pressure difference between upstream and downstream compartments; and $R_{j}$ is the resistance between compartments.

3 Pressure–Volume Relationships: each vascular compartment is assumed to be linearly compliant

(4) $$\begin{array}{c} P_{i}=\frac{V_{i}-V_{i,0}}{C_{i}} \end{array}$$

where i = PA, PC, and PV; $V_{i}-V_{0}$ is the difference between the stressed and the unstressed volume of the compartment; and $C_{i}$ is the compliance of the compartment.

The initial parameter set for each participant was derived from baseline physiological values combined with participant‐specific clinical measurements summarized in Table 2, which also provides a detailed description of the parameters fitted to RHC or echocardiographic data versus those derived directly from measurements or fixed to physiological values from the literature. The lumped‐parameter model was solved using MATLAB's stiff solver *ode15s* with adaptive time stepping. Simulations were run for 40 cardiac cycles to ensure stabilization, and all outputs were taken from the final cycle.

**TABLE 2:** Summary of the model initial conditions, parameters, definitions, and formula/value for calculations.

Initial conditions and parameters	Definition	Formula/value	References
*Initial conditions*			
$V_{0}^{\mathrm{PA}}$, mL	Unstressed pulmonary arterial volume	0.23SV	Brody et al. (1968)
$V_{0}^{\mathrm{PC}}$, mL	Unstressed pulmonary capillary volume	0.49SV	Brody et al. (1968)
$V_{0}^{\mathrm{PV}}$, mL	Unstressed pulmonary venous volume	0.28SV	Brody et al. (1968)
*Pressure estimates*			
$P_{\text{Mean}}^{\mathrm{RA}}$, mmHg	Mean right atrial pressure	RAP	RHC data
$P_{\mathrm{Sys}}^{\mathrm{RV}}$, mmHg	Right ventricular systolic pressure	$P_{\mathrm{Sys}}^{\mathrm{PA}}+0.5$	^a^
$P_{\mathrm{Dia}}^{\mathrm{RV}}$, mmHg	Right ventricular diastolic pressure	2.0	Hall et al. (2021)
$P_{\mathrm{Sys}}^{\mathrm{PA}}$, mmHg	Pulmonary arterial systolic pressure	$\max\left(\mathrm{PAP}\right)$	RHC data
$P_{\mathrm{Dia}}^{\mathrm{PA}}$, mmHg	Pulmonary arterial diastolic pressure	$\min\left(\mathrm{PAP}\right)$	RHC data
$P_{\text{Mean}}^{\mathrm{PA}}$, mmHg	Mean pulmonary arterial pressure	$\frac{2P_{\mathrm{Dia}}^{\mathrm{PA}}+P_{\mathrm{Sys}}^{\mathrm{PA}}}{3}$	Chemla et al. (2009)
$P_{\text{Mean}}^{\mathrm{PC}}$, mmHg	Mean pulmonary capillary pressure	$P_{\text{Mean}}^{\mathrm{PA}}-0.3\left(P_{\text{Grad}}\right)$	^b^
$P_{\text{Mean}}^{\mathrm{PV}}$, mmHg	Mean pulmonary venous pressure	$P_{\text{Mean}}^{\mathrm{PC}}-0.3\left(P_{\text{Grad}}\right)$	^b^
$P_{\text{Mean}}^{\mathrm{LA}}$, mmHg	Left atrial pressure	$\min\left(\text{PAWP},P_{\mathrm{Dia}}^{\mathrm{PA}}\right)$	RHC data
$P_{\text{Grad}}$, mmHg	Pressure gradient between mPAP and mPLA	$P_{\text{Mean}}^{\mathrm{PA}}-P_{\text{Mean}}^{\mathrm{LA}}$	^c^
*Parameter estimates*			
$R_{\mathrm{val},T}$, mmHg s/mL	Tricuspid valve resistance	$\frac{P_{\text{Mean}}^{\mathrm{RA}}-P_{\mathrm{Dia}}^{\mathrm{RV}}}{\mathrm{CO}}$	Colunga et al. (2023)
$R_{\mathrm{val},P}$, mmHg s/mL	Pulmonic valve resistance	$\frac{P_{\mathrm{Sys}}^{\mathrm{RV}}-P_{\mathrm{Sys}}^{\mathrm{PA}}}{\mathrm{CO}}$	Colunga et al. (2023)
$R_{\mathrm{PA}}$, mmHg s/mL	Pulmonary arterial resistance	$\frac{P_{\text{Mean}}^{\mathrm{PA}}-P_{\text{Mean}}^{\mathrm{PC}}}{\mathrm{CO}}$	Colunga et al. (2023)
$R_{\mathrm{PC}}$, mmHg s/mL	Pulmonary capillary resistance	$\frac{P_{\text{Mean}}^{\mathrm{PC}}-P_{\text{Mean}}^{\mathrm{PV}}}{\mathrm{CO}}$	Colunga et al. (2023)
$R_{\mathrm{PV}}$, mmHg/mL	Pulmonary venous resistance	$\frac{P_{\text{Mean}}^{\mathrm{PV}}-P_{\text{Mean}}^{\mathrm{LA}}}{\mathrm{CO}}$	Colunga et al. (2023)
$C_{\mathrm{PA}}$, mL/mmHg	Pulmonary arterial compliance	$\frac{V_{0}^{\mathrm{PA}}}{P_{\text{Mean}}^{\mathrm{PA}}}$	Colunga et al. (2023)
$C_{\mathrm{PC}}$, mL/mmHg	Pulmonary capillary compliance	$\frac{V_{0}^{\mathrm{PC}}}{P_{\text{Mean}}^{\mathrm{PC}}}$	Colunga et al. (2023)
$C_{\mathrm{PV}}$, mL/mmHg	Pulmonary venous compliance	$\frac{V_{0}^{\mathrm{PV}}}{P_{\text{Mean}}^{\mathrm{PV}}}$	Colunga et al. (2023)
$E_{\max}$, mmHg/mL	Maximum right ventricular elastance	$\frac{P_{\mathrm{Sys}}^{\mathrm{RV}}}{{\mathrm{ESV}}_{\mathrm{RV}}}$	Ellwein et al. (2008)
$E_{\min}$, mmHg/mL	Minimum right ventricular elastance	$\frac{P_{\mathrm{Dia}}^{\mathrm{RV}}}{{\mathrm{EDV}}_{\mathrm{RV}}}$	Ellwein et al. (2008)
$t_{\max}$, s	Right ventricular systolic duration	0.2 (CCD)	Ellwein et al. (2008)
$t_{\min}$, s	Right ventricular diastolic duration	0.5 (CCD)	Ellwein et al. (2008)

*Note:* $P_{\mathrm{Dia}}^{\mathrm{RV}}:$ A value of 2 mmHg was used as an initial guess for optimization. The final calibrated parameter value is determined from patient‐specific data. $P_{\text{Mean}}^{\mathrm{LA}}:$ Because only a single‐point PAWP measurement was available and no PAWP waveform was recorded, the left atrial pressure boundary condition was set to the lower of the reported PAWP or PA diastolic pressure. This choice ensures physiological consistency and avoids assigning a left atrial pressure greater than PA diastolic pressure.

Abbreviations: CCD, cardiac cycle duration; CO, cardiac output; EDV, end‐diastolic volume; mPAP, mean pulmonary artery pressure; mPLA, mean left atrial pressure; PA, pulmonary artery; PAP, pulmonary artery pressure; PAWP, pulmonary artery wedge pressure; PC, pulmonary capillary bed; PV, pulmonary venous bed; R, resistance; RV, right ventricle; SV, stroke volume; *V*_0_, initial/unstressed volume.

^a^ Used to ensure a slight pressure gradient across the pulmonary valve.

^b^ Assuming an equal pressure drop across the capillary and venous beds.

^c^ Calculated as the minimum of pulmonary artery wedge pressure (PAWP) and $P_{\mathrm{Dia}}^{\mathrm{PA}}$, reflecting the pressure constraints in the pulmonary venous and left atrial compartments based on clinical assumptions and model design.

### 2.4 Model Analysis and Calibration

**FIGURE 3:**
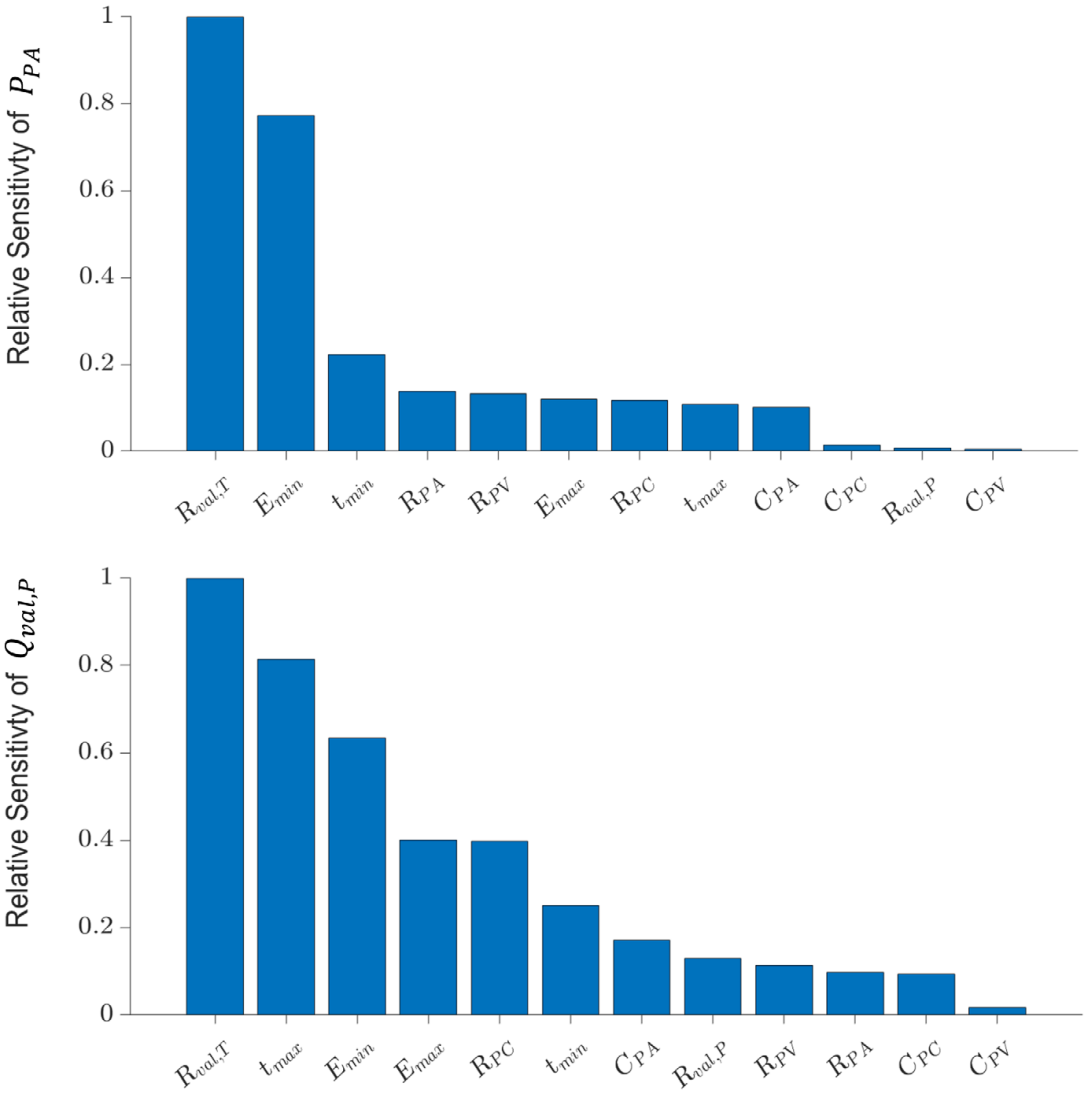
Sensitivity analysis of model parameters on pulmonary hemodynamics. Relative sensitivity of key model parameters on two primary output metrics: Pulmonary artery pressure ($P_{\mathrm{PA}}$) and blood flow across the pulmonary valve ($Q_{\mathrm{val},P}$). The top plot illustrates the sensitivity of $P_{\mathrm{PA}}$ to variations in model parameters, including resistances, compliances, and elastance, with each parameter's influence visualized along the x‐axis. The relative sensitivity is calculated by measuring the change in $P_{\mathrm{PA}}$ as each parameter is perturbed. The bottom plot shows the sensitivity analysis for $Q_{\mathrm{val},P}$, which represents the flow across the pulmonary valve into the PA, highlighting the most influential parameters on pulmonary flow. These sensitivity analyses provide insight into the model's robustness and identify the parameters that most significantly affect the outputs.

Sensitivity analyses were conducted to evaluate the relative influence of model parameters on $P_{\mathrm{PA}}$ and $Q_{\mathrm{val},P}$, which are the simulated quantities corresponding to the clinical data PAP and *Q*_RVOT_. We used the Morris' screening algorithm to systematically vary the resistance, compliance, and elastance parameters across a range of values. See references (Ellwein et al. 2008; Gerringer et al. 2018) for numerical details and implementation. This analysis provides insights into which parameters were most critical for accurate model calibration. Based on the results of the sensitivity analysis (Figure 3), $R_{\mathrm{val},P}$, $C_{\mathrm{PC}}$, and $C_{\mathrm{PV}}$ were identified as the least influential parameters on both $P_{\mathrm{PA}}$ and $Q_{\mathrm{val},P}$. As such, these parameters were excluded from the optimization process. Only the most influential parameters were made participant‐specific during the calibration process, reducing issues with parameter identifiability, reducing computational time, and improving the accuracy of subsequent clinical and model‐based analyses. The final set of influential parameters determined by sensitivity analysis was

(5) $$\begin{array}{c} \theta =\left\{R_{\mathrm{val},T},R_{\mathrm{PA}},R_{\mathrm{PC}},R_{\mathrm{PV}},C_{\mathrm{PA}},E_{\max},E_{\min},t_{\max},t_{\min}\right\}. \end{array}$$

When inferring these parameters, we ensure physiologically plausible bounds based on the baseline values, $\theta_{0}$. For all the parameters except $t_{\max}$ and $t_{\min}$, values were constrained to the interval between 1% and 600% of their respective baseline values. Timing parameters were constrained to fixed fractions of the cardiac cycle which $t_{\max}\in \left[0.05\;T,0.35\;T\right]$ and $t_{\min}\in \left[0.35\;T,0.7\;T\right]$ where T denotes the cycle length for each subject.

A custom cost function was designed to minimize residuals between model predictions and clinical measurements. The cost function included dynamic components (e.g., time‐resolved $P_{\mathrm{PA}}$ and $Q_{\mathrm{val},P}$ for the model, and PAP and *Q*_RVOT_ for the clinical data) and static data (e.g., maximum and minimum pressures, cardiac output). The cost function was calculated as:

(6) $$\begin{array}{c} J\left(\theta \right)=\sum_{j=1}^{N_{t}}W_{D}{\left(\frac{P_{\mathrm{PA}}\left(t_{j}\right)-\mathrm{PAP}\left(t_{j}\right)}{\max\left(\mathrm{PAP}\right)}\right)}^{2}+\sum_{j=1}^{N_{t}}W_{D}{\left(\frac{Q_{\mathrm{val},P}\left(t_{j}\right)-Q_{\text{RVOT}}\left(t_{j}\right)}{\max\left(Q_{\text{RVOT}}\right)}\right)}^{2}+W_{S}{\left(\frac{P_{\mathrm{PA},\min}-\mathrm{PA}P_{\min}}{\mathrm{PA}P_{\min}}\right)}^{2}+W_{S}{\left(\frac{P_{\mathrm{PA},\;\max}-\mathrm{PA}P_{\max}}{\mathrm{PA}P_{\max}}\right)}^{2} \end{array}+W_{S}{\left(\frac{Q_{\mathrm{val},P,\max}-Q_{\text{RVOT},\max}}{Q_{\text{RVOT},\max}}\right)}^{2}.$$

To account for both time series and static measurements important for qualitative assessment, we use weighted least squares with a dynamic‐data weight ($W_{D}=1/\sqrt{N_{t}}$, where $N_{t}$ is the number of time‐series points) and a static‐data weight ($W_{S}=2$) for static points. We note that maximum $P_{\mathrm{PA}}$, maximum $Q_{\mathrm{val},P}$, and minimum $P_{\mathrm{PA}}$—as well as their corresponding clinical counterparts—maximum PAP, maximum *Q*_RVOT_, and minimum PAP—are incorporated as separate components of the cost function so that these components are prioritized in the optimization procedure due to their significance.

We used the trust‐region reflective algorithm in the *lsqnonlin.m* function in MATLAB (Mathworks, Natick MA). This algorithm iteratively adjusted the parameters to minimize the cost function. To ensure robust parameter estimation, the optimization process was repeated using 20 different random perturbations of the initial parameter set. The best‐fit parameter set was then selected based on the minimum cost function value across all iterations. These parameters were used to simulate $P_{\mathrm{PA}}$ and $Q_{\mathrm{val},P}$ waveforms as well as RV PV loops.

After identifying the optimal parameter values, we simulated RV PV loops as well as other hemodynamic metrics. First, stroke work in the RV, ${\mathrm{SW}}_{\mathrm{RV}}$, was computed as the time integral of instantaneous ventricular pressure and the rate of change of volume over one cardiac cycle (i.e., the area inside the RV PV loop):

(7) $$\begin{array}{c} {\mathrm{SW}}_{\mathrm{RV}}=\int_{0}^{T}P\left(t\right)\dot{V}\left(t\right)\mathrm{dt} \end{array}$$

where $P\left(t\right)$ is the time‐varying right ventricular pressure, $V\left(t\right)$ is the corresponding instantaneous ventricular volume, $\dot{V}\left(t\right)$ is its temporal derivative, and T is the duration of the cardiac cycle.

### 2.5 Statistical Analysis

All hemodynamic data are presented as mean ± SD. For the simulations optimized to both $P_{\mathrm{PA}}$ and $Q_{\mathrm{val},P}$, as well as the predicted PV loops, *R* ^2^ values were calculated to assess the goodness‐of‐fit between model‐predicted waveforms and corresponding clinical data for each participant. Model‐derived RV PV loop metrics and phenotype group averages of RV function were computed and are reported as mean ± SD. Statistical comparisons between model‐predicted and clinical data were performed using paired two‐sample *t*‐tests for pre‐capillary PH and Ipc‐PH groups. A two‐tailed *p*‐value < 0.05 was considered statistically significant, with significance indicating poor agreement between model predictions and clinical measurements.

For model parameter estimates for the full dataset, comparisons across PH phenotypes were conducted using a Kruskal–Wallis test followed by a post hoc Dunn's test for pairwise comparisons, with *p* < 0.05 considered statistically significant. To assess group separability, Linear Discriminant Analysis (LDA) was performed using three feature sets: (1) clinical hemodynamic data, (2) model‐derived features, including model parameters and simulated PV loop metrics, and (3) a combined dataset integrating both clinical and model‐derived features. We visualized phenotype‐specific clustering in the reduced feature space to assess the separability across PH groups.

## 3 Results

### 3.1 Optimized $P_{\mathrm{PA}}$ and $Q_{\mathrm{val},P}$ Across PH Phenotypes

**FIGURE 4:**
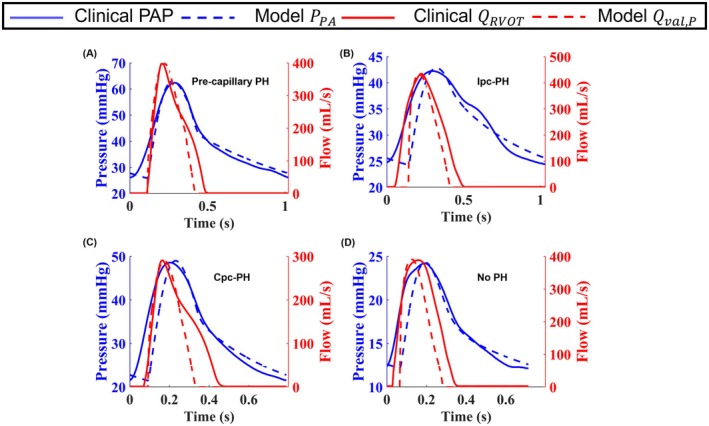
Time‐series pressure and flow fits for representative subjects from each phenotype are shown: (A) Pre‐capillary PH (#4), (B) Ipc‐PH (#11), (C) Cpc‐PH (#20), and (D) No PH (#23). Blue solid and dashed lines represent clinical and model‐predicted pulmonary arterial pressure, respectively, while red solid and dashed lines denote clinical and model‐RVOT flow.

**FIGURE 5:**
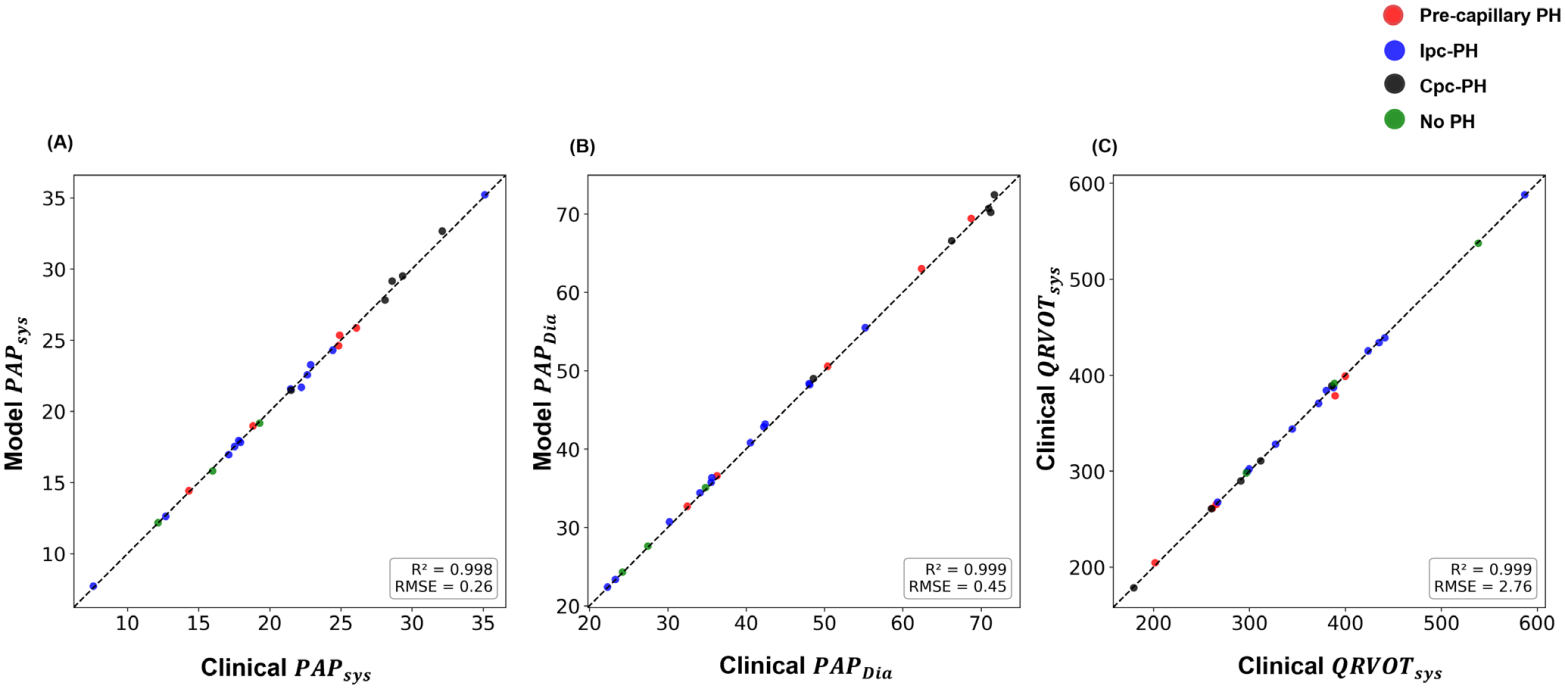
Parity plots comparing model‐predicted and measured values for (A) diastolic pressure, (B) systolic pressure, and (C) systolic flow. Each point represents an individual subject, color‐coded by phenotype: red = Pre‐capillary PH, blue = Ipc‐PH, black = Cpc‐PH, and green = No PH. The close alignment of data points along the identity line indicates strong model–data agreement, particularly for pressure metrics.

Time‐series PAP pressure and RVOT flow fits for a representative subject from each PH phenotype are illustrated in Figure 4, whereas Table 3 summarizes the average *R* ^2^ values across all PH phenotypes. As shown in Table 3, the model demonstrated consistently strong agreement between simulated and measured PAP pressure waveforms across all phenotypes, with *R* ^2^ values ranging from 0.78 ± 0.04 to 0.89 ± 0.04. Agreement for flow $\left(Q_{\mathrm{val},P}\right)$ was more variable, with the highest correlation observed in pre‐capillary PH (0.75 ± 0.14) and lower correlations in Ipc‐PH (0.48 ± 0.45). Cpc‐PH and No PH groups showed moderate flow agreement (0.66 ± 0.20 and 0.67 ± 0.18, respectively). Overall, pressure fits outperformed flow fits in all groups, indicating that the model captures pressure dynamics more reliably than flow waveforms. All participant pressure and flow fits are shown in the supplemental material (Figure S1). Figure 5 presents parity plots to evaluate agreement between model‐predicted and measured values. The model matches systolic and diastolic values in nearly all subjects.

**TABLE 3:** Model‐data agreement (*R* ^2^) for pressure and flow across PH phenotypes.

Phenotype	PAP *R* ^2^ (mean ± SD)	*Q*val,P *R* ^2^ (mean ± SD)
Pre‐capillary PH	0.89 ± 0.04	0.75 ± 0.14
Ipc‐PH	0.83 ± 0.14	0.48 ± 0.45
Cpc‐PH	0.87 ± 0.08	0.66 ± 0.20
No PH	0.78 ± 0.04	0.67 ± 0.18

### 3.2 RV PV Loop Simulation

**FIGURE 6:**
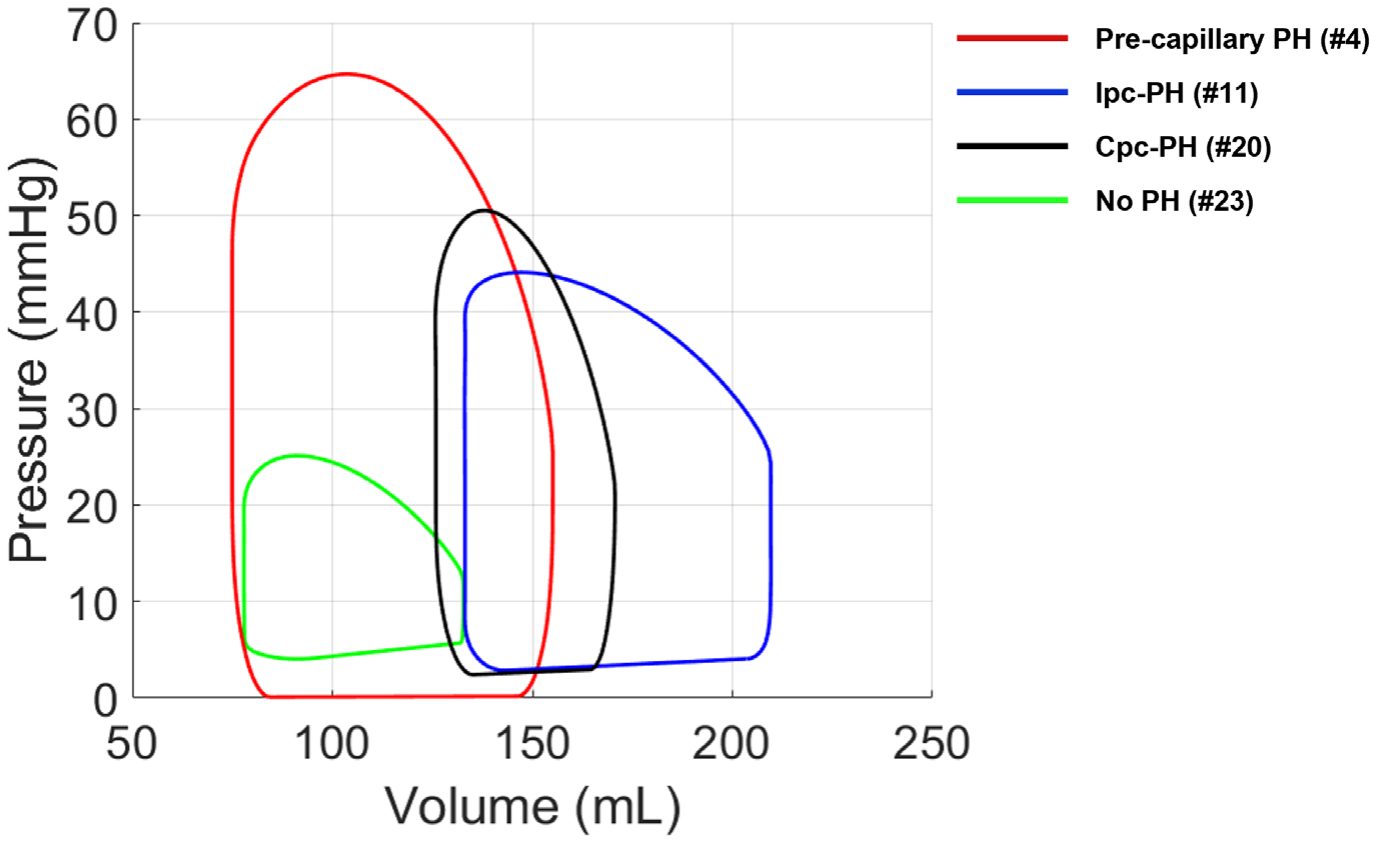
Simulated Right Ventricle (RV) PV loop data for representative subjects from each of the four phenotypes. Model‐derived PV loop metrics revealed distinct differences across groups red = Pre‐capillary PH, blue = Ipc‐PH, black = Cpc‐PH, and green = No PH.

Figure 6 shows simulated RV PV loop data for a representative subject from each of the four phenotypes. Simulated RV PV loops for all subjects are provided in the supplemental material (Figure S2). On average, model‐derived PV loop metrics revealed distinct differences among the four PH phenotypes (Table 4). As shown in Table 4, Ipc‐PH subjects demonstrated the largest ventricular volumes (EDV = 236 ± 132 mL; ESV = 178 ± 122 mL) but relatively low stroke work (0.24 ± 0.10 J) compared with Pre‐capillary PH and Cpc‐PH. Pre‐capillary PH subjects exhibited smaller volumes (EDV = 115 ± 35 mL; ESV = 68 ± 23 mL) and moderately higher stroke work (0.31 ± 0.20 mL‐mmHg). Cpc‐PH subjects had the smallest volumes (EDV = 96 ± 43 mL; ESV = 55 ± 41 mL) yet displayed higher end‐systolic pressure (ESP = 55 ± 9 mmHg) and stroke work (0.33 ± 0.12 mL mmHg), with ESP significantly greater than in Ipc‐PH and No PH (*p* < 0.05). The No PH group showed intermediate volumes (EDV = 106 ± 30 mL; ESV = 59 ± 19 mL) and the lowest stroke work (0.15 ± 0.05 mL mmHg), although this difference was not statistically significant.

**TABLE 4:** Model‐derived PV loop metrics (mean ± SD) across PH phenotypes.

Parameter	Pre‐capillary PH (mean ± SD)	Ipc‐PH (mean ± SD)	Cpc‐PH (mean ± SD)	No PH (mean ± SD)
EDV (mL)	115.1 ± 34.6	236.3 ± 131.6	95.6 ± 42.5	106.0 ± 30.3
ESV (mL)	68.4 ± 23.2	178.0 ± 121.6	54.8 ± 41.2	59.0 ± 19.4
SV (mL)	46.7 ± 17.0	58.2 ± 16.7	40.8 ± 12.3	47.0 ± 11.4
EDP (mmHg)	3.41 ± 2.71	5.88 ± 3.39	2.29 ± 0.79	4.35 ± 2.05
ESP (mmHg)	41.18 ± 11.34	33.18 ± 9.59	54.68 ± 9.03*†	25.15 ± 5.18
SW (J)	0.31 ± 0.20	0.24 ± 0.10	0.33 ± 0.12	0.15 ± 0.05

*Note:* Significance: *p < 0.05 vs. Ipc‐PH; †< 0.05 vs. No PH.

### 3.3 Model Parameter Estimates Across PH Phenotypes

**FIGURE 7:**
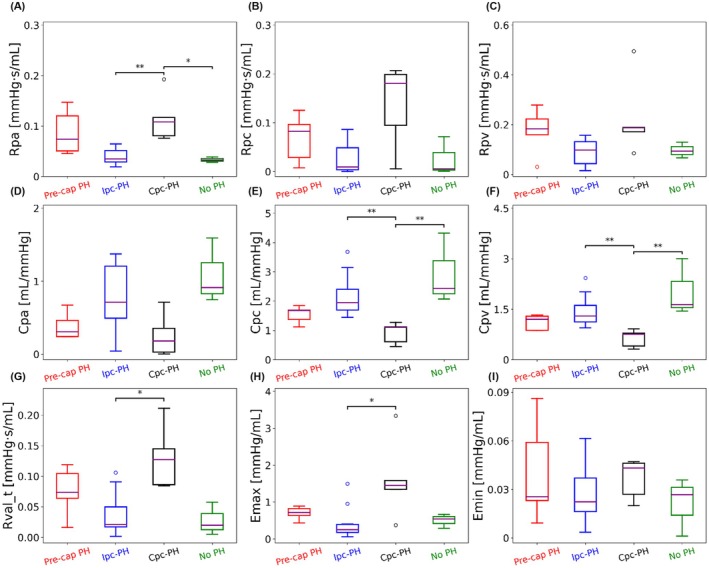
Comparison of model parameters across pulmonary hypertension phenotypes. (A) $R_{\mathrm{PA}}$ (pulmonary arterial resistance), (B) $R_{\mathrm{PC}}$(pulmonary capillary resistance), (C) $R_{\mathrm{PV}}$ (pulmonary venous resistance), (D) $C_{\mathrm{PA}}$ (pulmonary arterial compliance), (E) $C_{\mathrm{PC}}$ (pulmonary capillary compliance), (F) $C_{\mathrm{PV}}$ (pulmonary venous compliance), (G) $R_{\mathrm{val},T}$ (tricuspid valve resistance), (H) $E_{\max}$ (maximum elastance of the right ventricle) and (I 45) $E_{\min}$ (minimum elastance of the right ventricle) for all 25 participants: 5 Cpc‐PH, 12 Ipc‐PH, 5 pre‐capillary PH, and 3 No PH. Boxplots represent the median (purple line), interquartile range (boxes), and outliers (red plus signs). *p < 0.05, **p < 0.01 for inter‐group comparisons between PH phenotypes.

Model parameters are presented as averaged, phenotype‐grouped box‐and‐whisker plots in Figure 7. $R_{\mathrm{PA}}$ (pulmonary arterial resistance) was significantly greater in Cpc‐PH than either Ipc‐PH or No PH (*p* < 0.05). $R_{\mathrm{Pc}}$ (pulmonary capillary resistance) and $R_{\mathrm{PV}}$ (pulmonary venous resistance) were highest in Cpc‐PH, although the differences were not statistically significant. Also, $R_{\mathrm{val},T}$ was significantly higher in Cpc‐PH than Ipc‐PH (*p* < 0.05). $C_{\mathrm{PA}}$ was smallest in Cpc‐PH, although the difference was not statistically significant. In addition, $C_{\mathrm{PC}}$ and $C_{\mathrm{PV}}$ (neither included in the optimization process) were significantly lower in Cpc‐PH compared to either Ipc‐PH or No PH (*p* < 0.01 for both). Finally, $E_{\max}$ was significantly smaller in Ipc‐PH in comparison to Cpc‐PH (*p* < 0.05) whereas $E_{\min}$ tended to be highest in Cpc‐PH.

### 3.4 LDA Analysis

**FIGURE 8:**
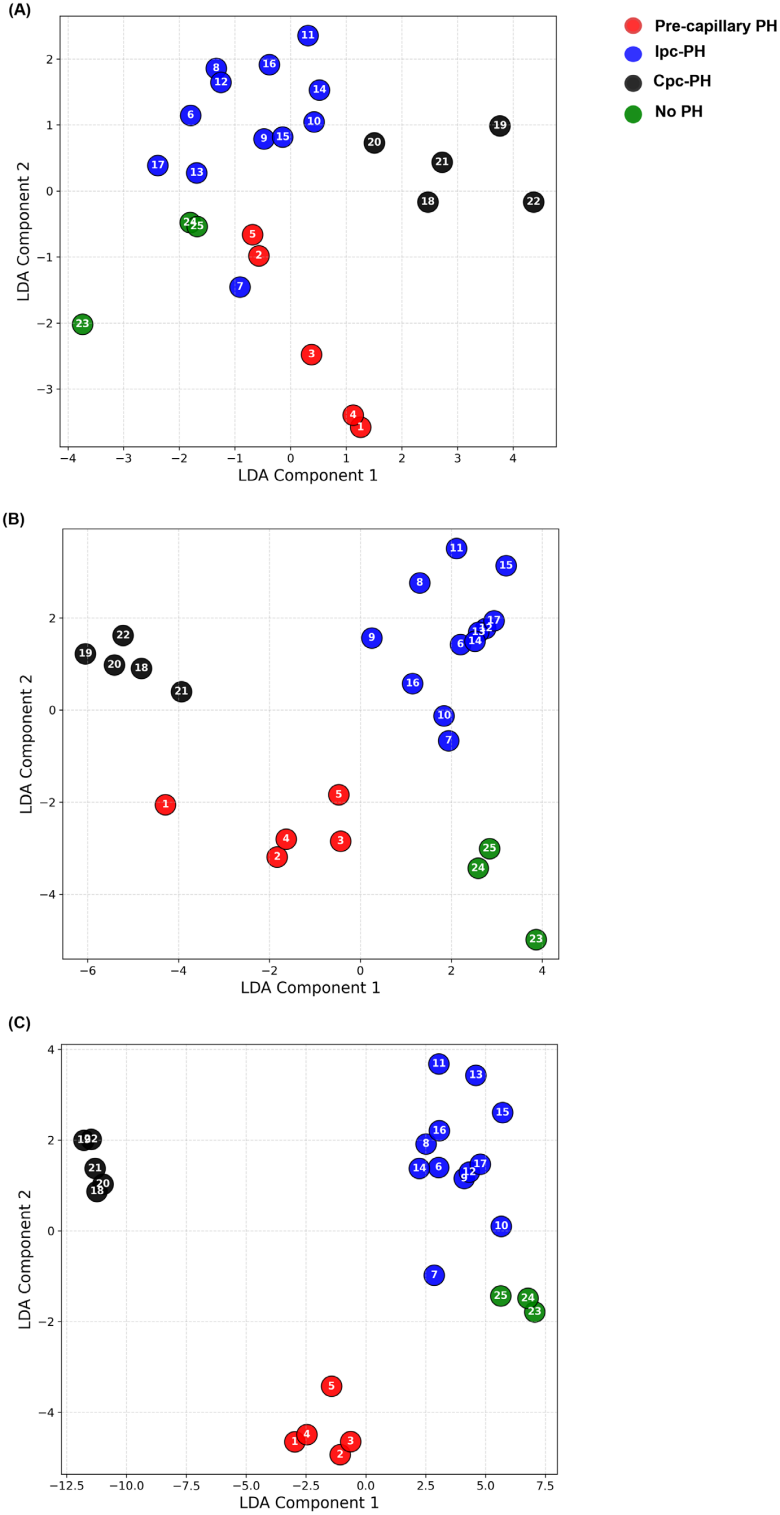
2D LDA projections illustrating phenotype separability using (A) clinical data, (B) model‐derived features, and (C) combined clinical and model features. The combined feature set shows the most distinct clustering across PH phenotypes: Pre‐capillary PH (red), Ipc‐PH (blue), Cpc‐PH (black), and No PH (green).

Visualization of LDA results is provided in Figure 8. When applied to clinical data (Figure 8A), the phenotypic clusters showed partial overlap, indicating limited separability. In contrast, LDA based solely on model‐derived features produced clearer clustering patterns, suggesting that simulated metrics capture additional physiological distinctions among groups (Figure 8B). The combined clinical and model feature set yielded the most distinct and well‐defined separation across phenotypes, highlighting the complementary value of integrating model‐derived information with clinical measurements for phenotype differentiation (Figure 8C).

## 4 Discussion

The goal of this study was to assess the accuracy and applicability of a lumped‐parameter model in quantifying vessel‐specific remodeling across different PH phenotypes and simulating RV PV loops. We hypothesized that participant‐specific models using routine clinical data could effectively replicate pulmonary vascular dynamics and RV function. Our findings support this hypothesis in a small cohort (*n* = 25) representing four distinct PH phenotypes. Key findings from this study include: (1) across the entire cohort, the lumped‐parameter model demonstrated reasonable agreement with clinical data; (2) PH subtypes showed distinct differences in vessel‐specific remodeling, with Cpc‐PH subjects exhibiting the highest resistances and the lowest compliances among all groups, reflecting a stiffer and less compliant pulmonary circulation. In contrast, Ipc‐PH showed the lowest resistance and highest proximal compliance, while No PH subjects demonstrated the greatest mid‐ and distal compliances, indicative of preserved vascular elasticity; (3) the model‐derived PV loop metrics showed that ventricular volumes and pressures varied across phenotypes, with Cpc‐PH exhibiting the highest pressure and stroke work, Ipc‐PH the largest volumes, and No PH the lowest overall workload. Finally, (4) LDA analysis showed qualitatively separable patterns between phenotypes when using model simulations, suggesting that model‐derived, nonmeasurable parameters provide additional discriminatory information (Figure 8). These findings highlight the potential of lumped‐parameter models for less invasive RV assessment in PH, while emphasizing the need for further refinement, particularly for non‐PH participants.

In the context of current PH research, our study builds upon recent efforts to develop less invasive approaches for assessing RV function and pulmonary vascular dynamics (Kheyfets et al. 2016; Tang et al. 2020; Kim et al. 2023; Gerringer et al. 2018; Harrod et al. 2021). Our study demonstrates a simple method to simulate PV loop data with the long‐term goal of developing a completely noninvasive tool for PH assessment. Gerringer et al. demonstrated that lumped‐parameter models can simulate PAH progression by capturing key hemodynamic changes such as PVR and compliance. While their model was calibrated to time‐dependent data, it lacked direct information about the RV, pulmonary capillaries, and pulmonary veins (Gerringer et al. 2018). We calibrated model parameters using pressure and flow data from different modalities, collected during the same clinical encounter. This simultaneity minimizes physiological variability between tests and improves the reliability and interpretability of parameter estimates. Our model captured the measured PAP and QRVOT waveforms in most subjects with reasonable accuracy while incorporating information about the arterial, capillary, and venous compartments of the pulmonary circulation as well as the RV (Table 3, Figure 4). Although the time‐series waveforms were not perfectly aligned in some subjects, the model preserved the systolic and diastolic features, which are the most clinically relevant aspects (Figure 5). Similarly, Tang et al. developed a lumped‐parameter model to simulate hemodynamic changes in specific PH etiologies, including distal pulmonary artery stenosis, left ventricular diastolic dysfunction, ventricular septal defect, and mitral stenosis, but their model also lacked participant‐specific calibration (Tang et al. 2020). Our study improves upon this approach by introducing subject‐specificity. Harrod et al. (2021) explored predictive modeling in secondary PH due to left ventricular dysfunction using a lumped‐parameter model, which was calibrated with clinical data. Lastly, Kheyfets et al. developed a zero‐dimensional model for PH based on RHC data from 115 pediatric subjects, including *P*_max_, *P*_min_, mPAP, CO, and stroke volume, without dynamic waveform information (Kheyfets et al. 2016). This limited data restricts the model's ability to capture the full complexity of RV function and the pulmonary circulation, unlike our study, which uses a more comprehensive time‐series informed dataset to provide a more accurate description of RV function.

Recent studies have proposed several approaches to reconstruct RV pressure–volume loops directly from routine clinical data. Richter et al. (2022) generated noninvasive PV loop predictions by combining 3D echocardiographic RV volumes with estimated pressure curves, while Kremer et al. (2025) created RV PV loops from routine RHC by estimating RV volumes from pressure waveforms with external calibration. Also, Kiarad et al. (2024) constructed intraoperative PV loops by synchronizing high‐fidelity RV pressure recordings with 3D echocardiographic volumes. In contrast, our PV loops are model‐derived outputs from a simple lumped‐parameter framework calibrated to routine clinical measurements, dynamic PA pressure and RVOT flow, without requiring RV volume data or specialized imaging. Beyond loop shape, our model provides mechanistic estimates of pulmonary vascular resistance and compliance components, and valve resistance, enabling quantitative PH phenotyping that is not accessible through reconstruction‐based PV loop methods alone.

Patient‐specific simulated PV loops across the four PH phenotypes are presented in Figure 6 and summarized in Table 4. Overall, the model‐derived PV loop metrics highlight distinct right ventricular functional profiles across PH phenotypes. Both Ipc‐PH and Cpc‐PH had increased end‐systolic volumes compared to Pre‐cap and no PH. Due to increased pulmonary afterload in Cpc‐PH, stroke volume was reduced as reflected by lower end‐diastolic volume (homeometric adaptation), while in Ipc‐PH, stroke volume remained preserved via higher end‐diastolic volume (heterometric adaptation). When comparing model predictions between PH subjects and the No PH group, PH subjects exhibited higher pressures, larger ventricular volumes (except for Cpc‐PH, which showed relatively smaller volumes), and greater stroke work, consistent with the increased hemodynamic load and compensatory contractile response associated with PH (Csósza et al. 2024). We note that while the absolute volumes generated by simulated RV PV‐loops are less reliable, the predicted stroke work performed by the RV is more accurate and provides insights into abnormal myocardial energetics and increased oxygen consumption (Brener et al. 2022; Suga 1990; Seemann et al. 2019). The SW trends (Table 4) among the four hemodynamic groups revealing a trend in the order of increasing pulmonary vascular disease (No PH < Ipc‐PH < pre‐capillary PH < CpcPH), consistent with prior literature (Caravita et al. 2018). Collectively, these results suggest that the model captures phenotype‐specific differences in ventricular loading and performance, providing quantitative insight into underlying pathophysiological mechanisms.

The parameter estimates across PH phenotypes reveal distinct differences, particularly in Cpc‐PH, where higher values of $R_{\mathrm{val},P}$, $R_{\mathrm{val},T}$ $R_{\mathrm{PA}}$, $R_{\mathrm{PV}}$, $E_{\max}$, and $E_{\min}$ and lower values of $C_{\mathrm{PA}}$, $C_{\mathrm{PC}}$, and $C_{\mathrm{PV}}$ were observed compared to Ipc‐PH and No PH (Figure 7). As described in the Methods section, the valves are modeled as diodes (loss‐less one‐way switches); therefore, the valve resistance parameters ($R_{\mathrm{val},P}$ and $R_{\mathrm{val},T}$) represent effective inflow and outflow resistances rather than true physical narrowing or intrinsic valve disease. These differences reflect the pathophysiological characteristics of Cpc‐PH, where both pre‐ (PA) and postcapillary (PV) remodeling contribute to altered impaired RV function and RV‐PA uncoupling (Humbert et al. 2022). The model's ability to capture these alterations highlights its potential to provide valuable insights into the pulmonary vascular and RV dysfunction in this phenotype. Importantly, the model provides estimates of vascular resistances, compliances, valve resistances, and RV contractility, which are not directly measured in routine clinical assessments. These estimates not only offer additional mechanistic insights into PH‐related hemodynamic changes but also enable differentiation between Cpc‐PH, Ipc‐PH, and No PH, providing a more refined understanding of PH phenotype. In contrast, no significant differences were found between pre‐capillary PH and the other phenotypes. This result may be due to the relatively simplistic model or small and heterogenous pre‐capillary PH cohort. Future model refinements—such as incorporating nonlinear resistance and compliance or simulating dynamic conditions like exercise—may enhance the model's capability to differentiate more PH phenotypes.

It is important to distinguish between different types of PH, as each phenotype has distinct underlying mechanisms and requires a different therapeutic approach (Rose‐Jones and Mclaughlin 2015). To explore this, we applied LDA using three different feature combinations. As shown in Figure 8, clinical data alone lacked sufficient discriminatory resolution to separate phenotypes with subtle physiological differences. However, when clinical features were combined with model‐derived information, the overall separability between phenotypes improved markedly. This finding suggests that incorporating hemodynamic modeling such as a simple lumped parameter model can provide additional mechanistic insights that are not captured through clinical measurements alone.

Several limitations warrant consideration. First, the small sample size within each PH phenotype limits the generalizability of our findings and restricts the statistical power to detect subtle differences in pulmonary hemodynamic metrics. Furthermore, a larger sample size would make the LDA analysis more robust. Second, the simplified lumped‐parameter model used in this study does not fully capture the complexity of RV‐PA interactions, nor does it consider pulse‐wave propagation through the pulmonary vasculature. Third, in this study, we used an open‐loop system, which does not closely reflect physiological reality. However, a closed loop approach would significantly increase the model's complexity, introduce additional parameters, and make parameter inference more challenging and computationally intensive. Moreover, to achieve our goal of using a simple model to understand how pulmonary hypertension affects vessel‐specific remodeling and RV function, the open loop approach was sufficient. Finally, the present study employs a linear elastance model, which doesn't capture the nonlinear end‐diastolic pressure volume relationship seen in vivo. Future applications of this model with a nonlinear elastance function may better identify RV diastolic dysfunction with PH.

## 5 Conclusion

In summary, this study successfully calibrated a lumped‐parameter model to participant‐specific RHC and echocardiography data across different PH phenotypes. We show that the model replicates measured clinical data and predicts pressure–volume loop dynamics, which helps bypass the need for invasive PV loop acquisition and provides information useful for distinguishing between different PH phenotypes. These results highlight the potential of lumped‐parameter models as a reliable tool for assessing vessel‐specific remodeling and RV function. While further refinement is needed, especially in simulating hemodynamics in non‐PH participants, modeling and simulation could improve management of PH. Although the present study relies on invasively measured PA pressure waveforms for model calibration, future work will focus on extending the framework to incorporate noninvasive pressure estimates. Echo‐derived PA pressure and Doppler‐based flow measurements could enable fully noninvasive model calibration, although additional strategies will be needed to address measurement noise and parameter identifiability. Demonstrating robust performance using noninvasive inputs would substantially enhance the clinical utility and scalability of the model. Future efforts will also extend the framework to dynamic conditions, such as exercise.

## Author Contributions

The authors confirm contribution to the paper as follows: study conception and design: C.G.L., M.J.C., and N.C.C.; data collection: F.R. and C.E.K.; analysis and interpretation of results: A.K., C.G.L., F.R., M.J.C., and N.C.C.; draft manuscript preparation: A.K., C.G.L., M.J.C., F.R., and N.C.C. All authors reviewed the results and approved the final version of the manuscript.

## Funding

This study was supported from the National Center for Advancing Translational Sciences, Grant/Award Number: KL2TR002374‐07 (FR), American Heart Association, Grant/Award Number: 23CDA1057697 (FR), the NIH T32HL116270 (CGL), NIH R01HL154624 (NCC), and NIH R01HL147590 (NCC). MJC was supported by the National Center for Research Resources and the National Center for Advancing Translational Sciences, National Institutes of Health, through Grant *TL1001415*. The content is solely the responsibility of the authors and does not necessarily represent the official views of the NIH.

## Conflicts of Interest

The authors declare no conflicts of interest.

## Supporting information


**Figure S1:** Optimized $P_{\mathrm{PA}}$ and $Q_{\mathrm{val},P}$ waveforms. Optimized $P_{\mathrm{PA}}$ and $Q_{\mathrm{val},P}$ waveforms (dashed lines) are presented with clinical data (solid lines) for all participants grouped by PH phenotype: (A) Pre‐capillary PH (*n* = 5), (B) Ipc‐PH (*n* = 12), (C) Cpc‐PH (*n* = 5), and (D) No PH (*n* = 3).
**Figure S2:** Optimized $P_{\mathrm{PA}}$ and $Q_{\mathrm{val},P}$ waveforms. Simulated RV PV loops for all participants grouped by PH phenotype: (A) Pre‐capillary PH (red), (B) Ipc‐PH (blue), (C) Cpc‐PH (black), and (D) No PH (green).

## Data Availability

The data that support the findings of this study are available on request from the corresponding author. The data are not publicly available due to privacy or ethical restrictions.
