# Supplementary material for: Patient‐Specific Lumped‐Parameter Model for Quantifying Vessel‐Specific Remodeling and Predicting Right Ventricular Function in Pulmonary Hypertension
Source: Compr Physiol. 2026 Jan 26;16(1):e70102. doi: 10.1002/cph4.70102 (PMC12835583; doi:10.1002/cph4.70102)
Supplement: Supplementary file 1 — Figure S1: Optimized PPA and Qval,P waveforms. Optimized PPA and Qval,P waveforms (dashed lines) are presented with clinical data (solid lines) for all participants grouped by PH phenotype: (A) Pre‐capillary PH (n = 5), (B) Ipc‐PH (n = 12), (C) Cpc‐PH (n = 5), and (D) No PH (n = 3). Figure S2: Optimized PPA and Qval,P waveforms. Simulated RV PV loops for all participants grouped by PH phenotype: (A) Pre‐capillary PH (red), (B) Ipc‐PH (blue), (C) Cpc‐PH (black), and (D) No PH (green). [file CPH4-16-e70102-s001.docx]

**Supplemental Material to: Patient-Specific Lumped-Parameter Model for Quantifying Vessel-Specific Remodeling and Predicting Right Ventricular Function in Pulmonary Hypertension**

**Author List**: Christopher G. Lechuga, PhD^1†^; Amirreza Kachabi, MS^1†^, Mitchel J. Colebank, PhD^1,3^; Claudia E. Korcarz, DVM, RDCS^2^; Farhan Raza, MD^2^; Naomi C. Chesler, PhD^1^

**Affiliations: ^1^**Edwards Lifesciences Foundation Cardiovascular Innovation and Research Center (CIRC) and Department of Biomedical Engineering, University of California, Irvine, Irvine, CA; ^2^ Department of Medicine-Cardiovascular Division, University of Wisconsin – Madison, Madison, WI; ^3^Department of Mathematics and Department of Biomedical Engineering, University of South Carolina;

^†^ These two authors contributed equally.

**Corresponding author:** Naomi C. Chesler, Email: [nchesler@uci.edu](mailto:nchesler@uci.edu), Office Address: 6830 ISEB; 419 S. Circle Drive, Samueli School of Engineering, University of California, Irvine, Irvine, CA 92697

**Author Contributions:** The authors confirm contribution to the paper as follows: study conception and design: CGL, MJC, NCC; data collection: FR, CEK; analysis and interpretation of results: AK, CGL, FR, MJC, NCC; draft manuscript preparation: AK, CGL, MJC, FR, NCC. All authors reviewed the results and approved the final version of the manuscript.

**Sources of Funding:** This study was supported from the National Center for Advancing Translational Sciences, Grant/Award Number: KL2TR002374‐07 (FR), American Heart Association, Grant/Award Number: 23CDA1057697 (FR), the NIH T32HL116270 (CGL), NIH R01HL154624 (NCC), and NIH R01HL147590 (NCC). MJC was supported by the National Center for Research Resources and the National Center for Advancing Translational Sciences, National Institutes of Health, through Grant TL1001415. The content is solely the responsibility of the authors and does not necessarily represent the official views of the NIH.

1. **Optimized** $\boldsymbol{P}_{\boldsymbol{PA}}$ **&** $\boldsymbol{Q}_{\boldsymbol{val,P}}$ **Across PH Phenotypes**


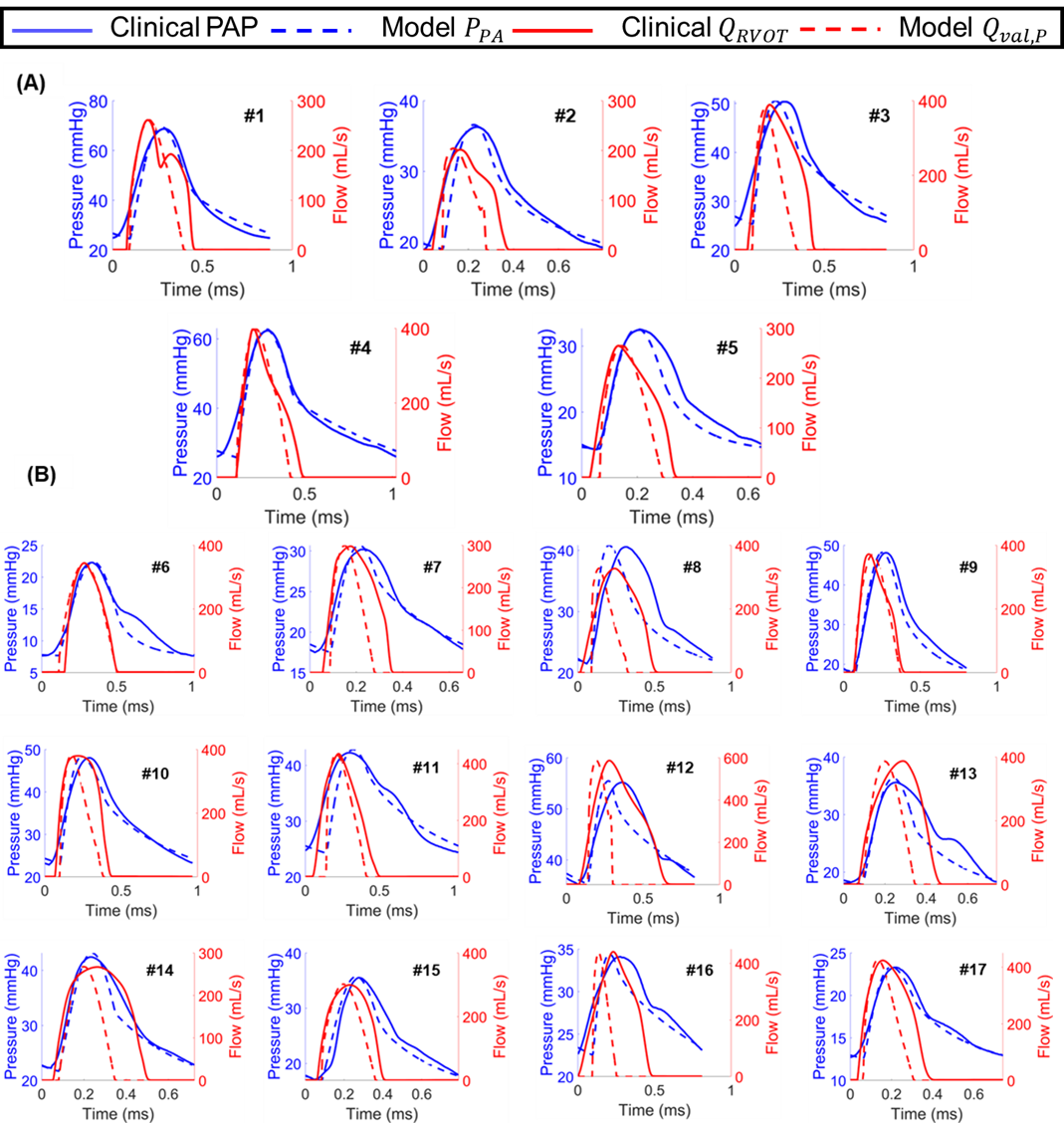


**Figure S1:** **Optimized** $\boldsymbol{P}_{\boldsymbol{PA}}$ **and** $\boldsymbol{Q}_{\boldsymbol{val,P}}$ **waveforms.** Optimized $P_{PA}$ and $Q_{val,P}$ waveforms (dashed lines) are presented with clinical data (solid lines) for all participants grouped by PH phenotype: (**A**) Pre-capillary PH (n = 5), (**B**) Ipc-PH (n = 12), (**C**) Cpc-PH (n = 5), and (**D**) No PH (n = 3).


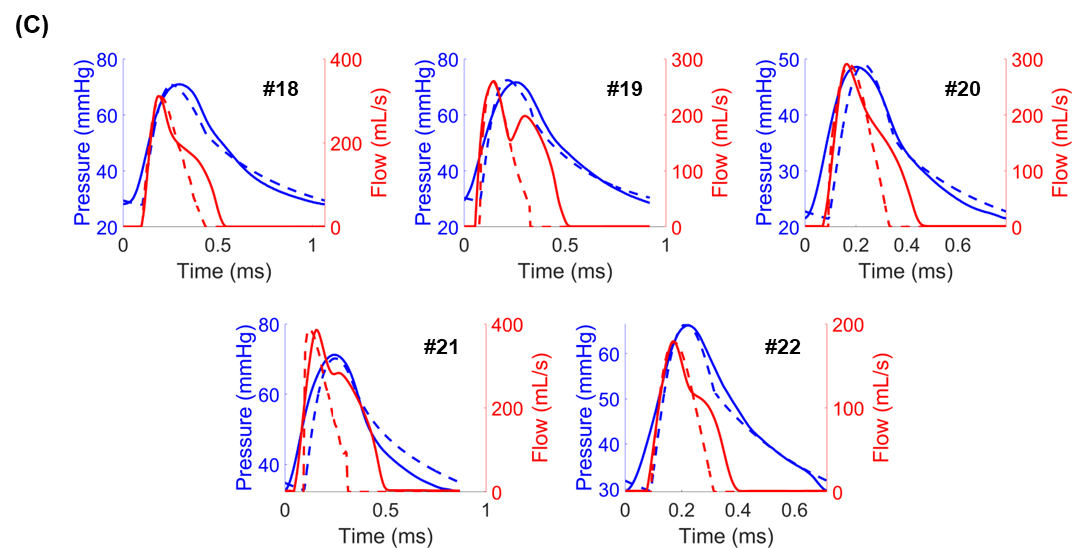

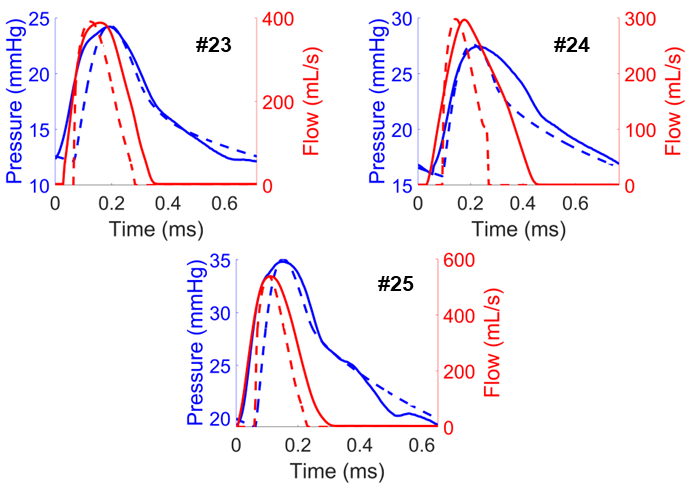

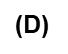


1.
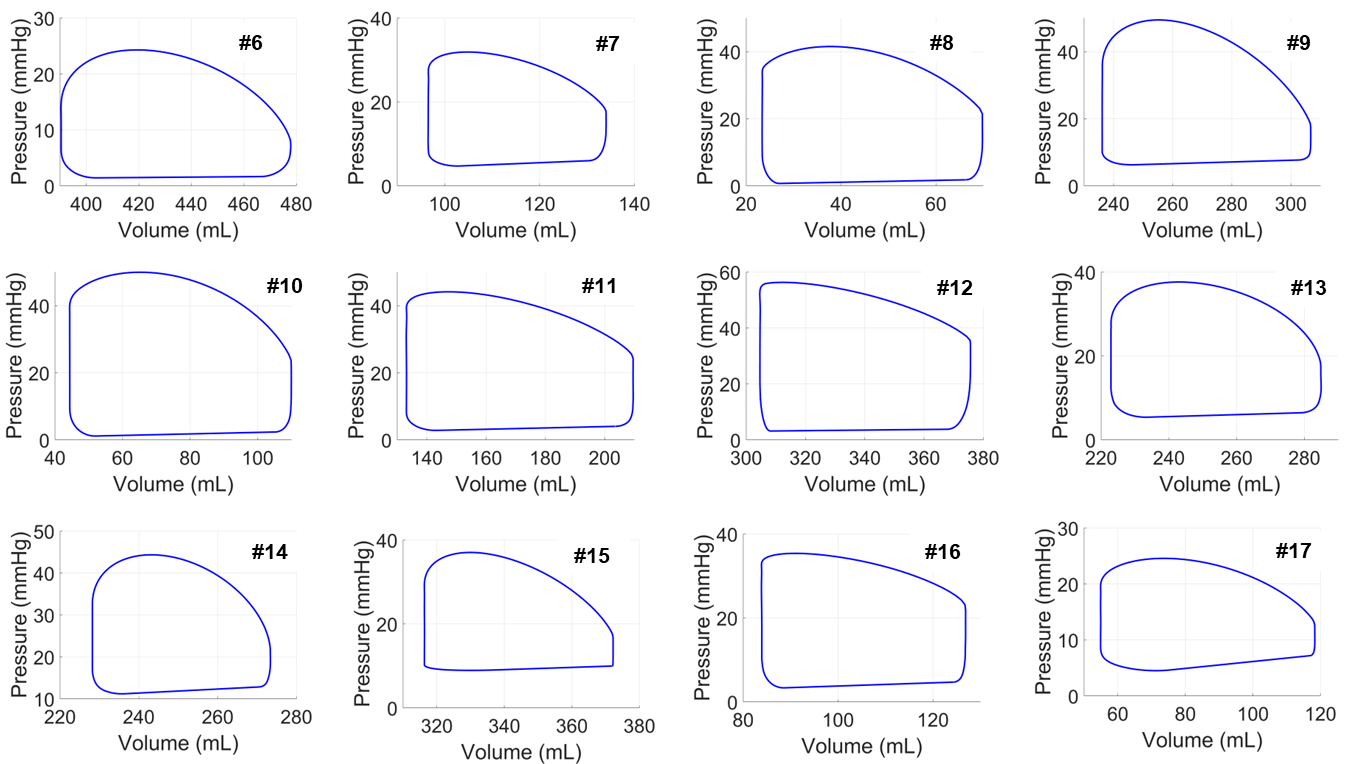

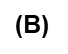

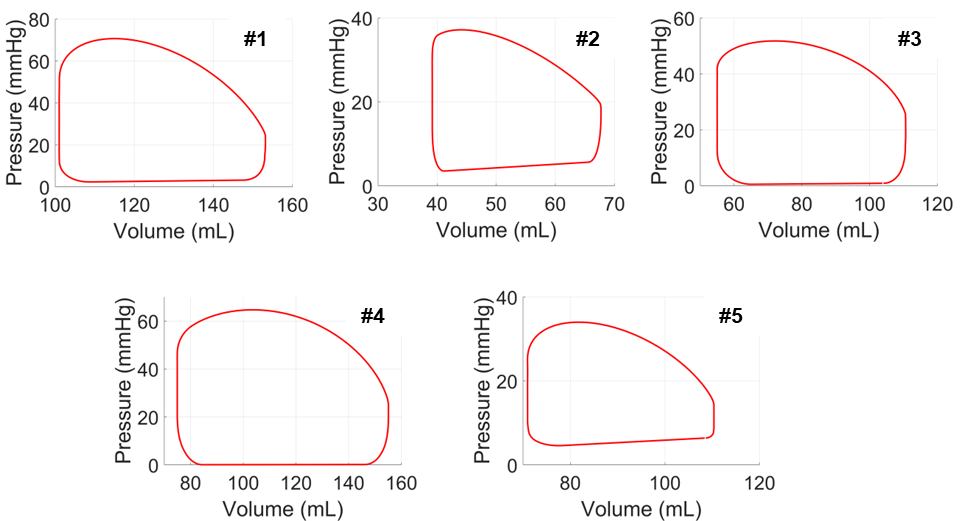

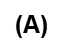
**Simulated RV PV loops** **Across PH Phenotypes**


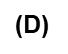

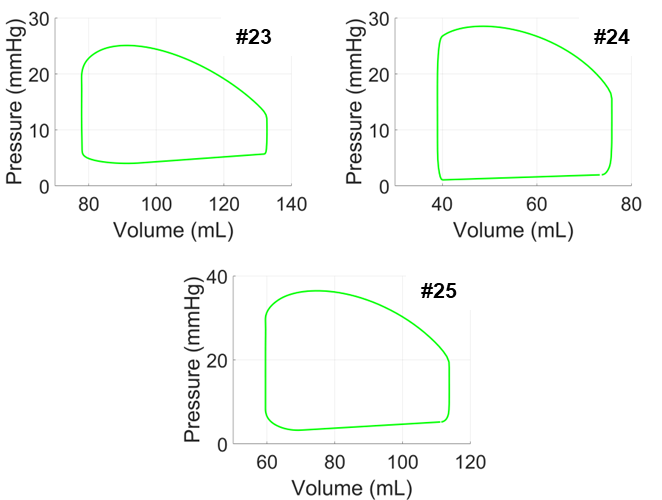

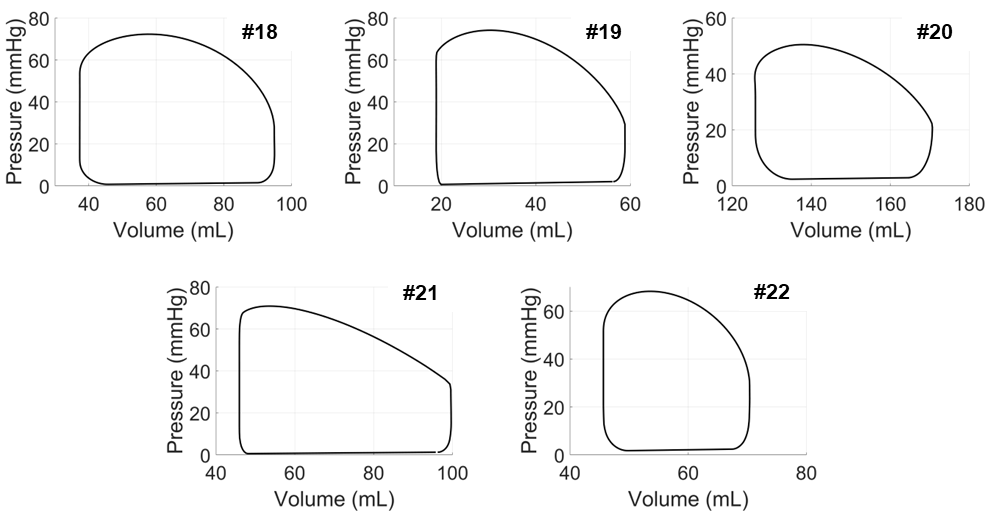

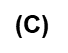


**Figure S2: Optimized** $\boldsymbol{P}_{\boldsymbol{PA}}$ **and** $\boldsymbol{Q}_{\boldsymbol{val,P}}$ **waveforms.** Simulated RV PV loops for all participants grouped by PH phenotype: (**A**) Pre-capillary PH (red), (**B**) Ipc-PH (blue), (**C**) Cpc-PH (black), and (**D**) No PH (green).
